# Supplementary material for: Thrombopoiesis is spatially regulated by the bone marrow vasculature
Source: Nat Commun. 2017 Jul 25;8:127. doi: 10.1038/s41467-017-00201-7 (PMC5527048; doi:10.1038/s41467-017-00201-7)
Supplement: Supplementary file 1 — Supplementary Information [file 41467_2017_201_MOESM1_ESM.pdf]

File Name: Supplementary Information
Description: Supplementary Figures.

File Name: Supplementary Movie 1
Description: Representative two-photon intra-vital microscopy recording of MKs (green, GPIX) in a naïve mouse, which did not display significant movement during the measurement time of over 3 h. The vasculature is depicted in red (dextran, anti-CD105). A vessel-associated MK (upper cell), and a non-vessel-associated MK (lower cell) are shown. Scale bar 15 µm.

File Name: Supplementary Movie 2
Description: Optical slicing by LSFM of intact sternal bone marrow leading to 3D reconstruction (grid size 100 µm) with blood vessels (red, CD105) and MKs (green, GPIX).

File Name: Supplementary Movie 3
Description: MKs (green, GPIX) and blood vessels (red, CD105) in reconstructed bone marrow (BM) of the femur (grid size 100 µm).

File Name: Supplementary Movie 4
Description: MKs (green, GPIX) and blood vessels (red, CD105) in reconstructed bone marrow (BM) of the sternum (grid size 100 µm).

File Name: Supplementary Movie 5
Description: MKs (green, GPIX) and blood vessels (red, CD105) in reconstructed bone marrow (BM) of the femur diaphysis. The autofluorescence-derived bone structures are depicted in grey color. Grid size 200 µm.

File Name: Supplementary Movie 6
Description: MKs (green, GPIX) and blood vessels (red, CD105) in reconstructed bone marrow (BM) of the femur epiphysis. The autofluorescence-derived bone structures are depicted in grey color. Grid size 200 µm.

File Name: Supplementary Movie 7
Description: Optical slicing by LSFM of intact femural bone marrow (femur head) leading to 3D reconstruction (grid size 200 µm) with blood vessels (red, CD105) and megakaryocytes (green, GPIX). The bone marrow is depicted in yellow (autofluorescence), while bone is displayed in light grey. Please note the presence of MK-rich BM within the entire inter-bone space.

File Name: Supplementary Movie 8
Description: MKs (green, GPIX) and blood vessels (red, CD105) in reconstructed bone marrow (BM) of the sternum. The autofluorescence-derived bone structures are depicted in grey color. Grid size 200 µm.

File Name: Supplementary Movie 9
Description: MKs (green, GPIX) and blood vessels (red, CD105) in reconstructed bone marrow (BM) of the skull. The autofluorescence-derived bone structures are depicted in grey color. Grid size 200 µm.

File Name: Supplementary Movie 10
Description: Two representative two-photon microscopy recordings of MKs (green, GPIX) of a thrombocytopenic mouse. The first example shows a vessel-associated MK that remained sessile for 2.5 h before starting to form proplatelets in a vessel (red, CD105) (see also Fig. 5), the second example a non-vessel-associated MK.

File Name: Peer Review File
Description:

Supplementary Figures

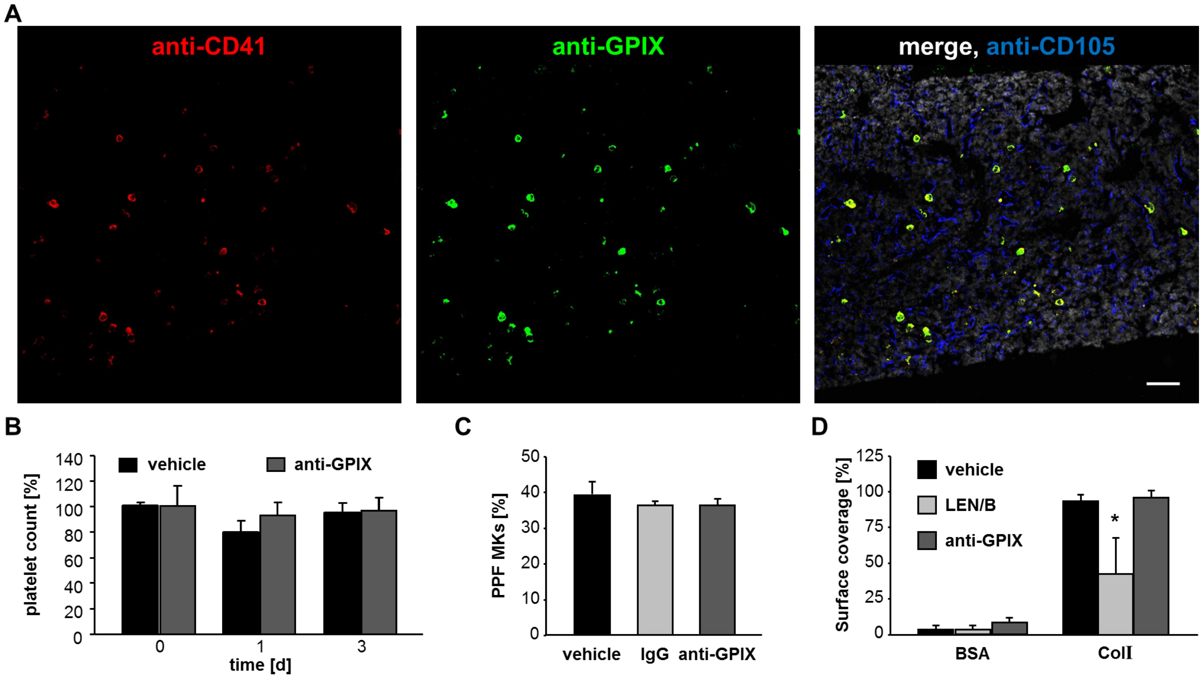

Supplementary Fig. 1. The anti-GPIX derivative used for intra-vital imaging stains all megakaryocytes without affecting platelet production. **(A)** Virtually all megakaryocytes (MKs, CD41+, red) in cryo-sections of femur are stained by the used anti-GPIX derivative (green). Vessels (CD105+, blue) and nuclei (DAPI, grey) are depicted in the merged image, scale bar: 100 µm. **(B)** Male C57Bl/6J mice received either 1.5 µg Alexa488-conjugated anti-GPIX derivative/g body weight (grey bars) or vehicle (black bars) and platelet counts were determined by flow cytometry. Depicted are mean ± standard deviation of relative platelet counts (compared to day 0); n=5. **(C)** Proplatelet formation (PPF) of fetal-liver derived MKs on BSA was assessed in the presence of vehicle, control IgG or the anti-GPIX derivative (10 µg/ml). Bar graphs represent mean ± SD; n=4. **(D)** Surface coverage of fetal-liver derived MKs on BSA or collagen I (col I) was assessed in the presence of vehicle, the anti-GPIX derivative (10 µg/ml) or the α2β1-blocking antibody LEN/B. Bar graphs represent mean ± SD; n=4. *, $p<0.05$ (Mann-U-Whitney test).

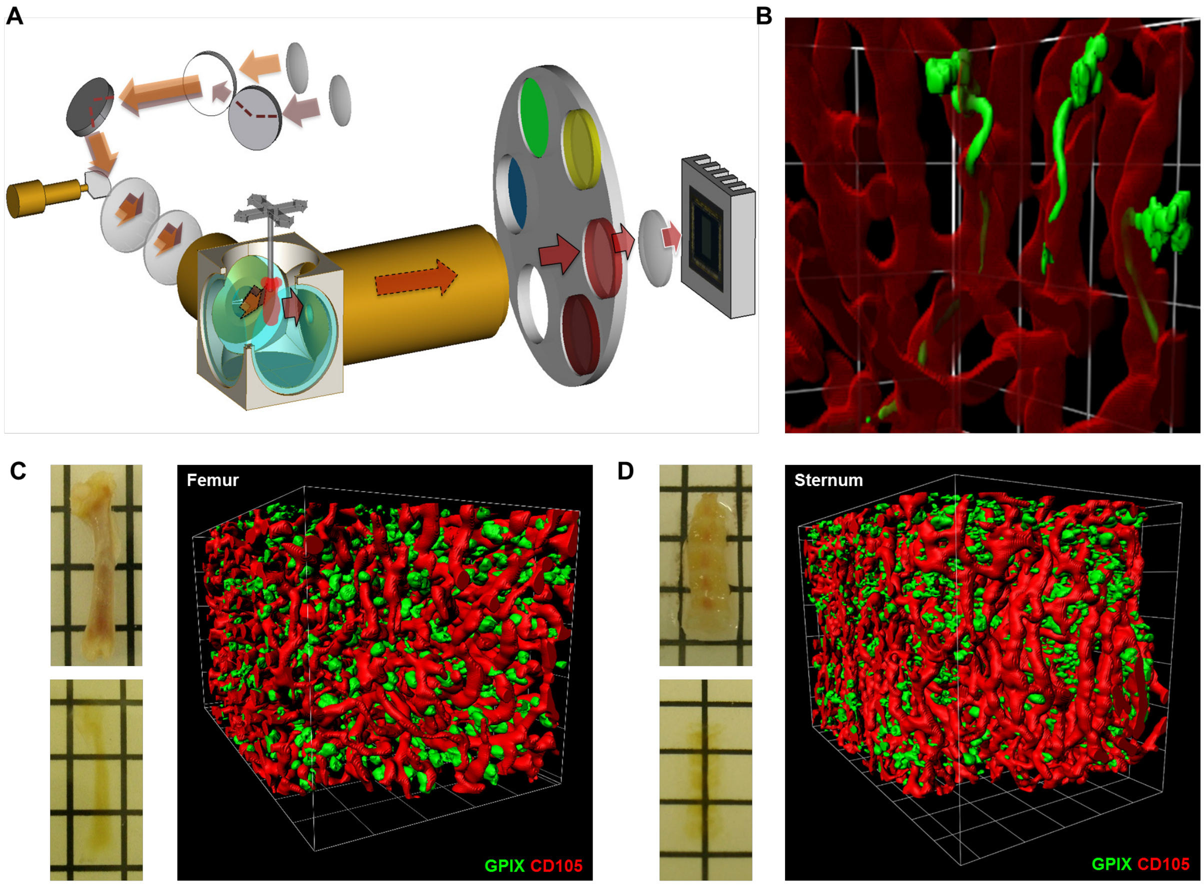

Supplementary Fig. 2. LSFM imaging reveals a dense BM vasculature. **(A)** The home-built LSFM setup allows image acquisition at a resolution sufficient to depict proplatelets **(B)**. **(C-D)** Optical clearing of intact bones and subsequent LSFM-analysis shows the dense blood vessel network (red, CD105) in femur **(C)** and sternum **(D)** and reveals a homogeneous 3D distribution of MKs (green, GPIX) in the BM. Unit size for all images: 100 µm.

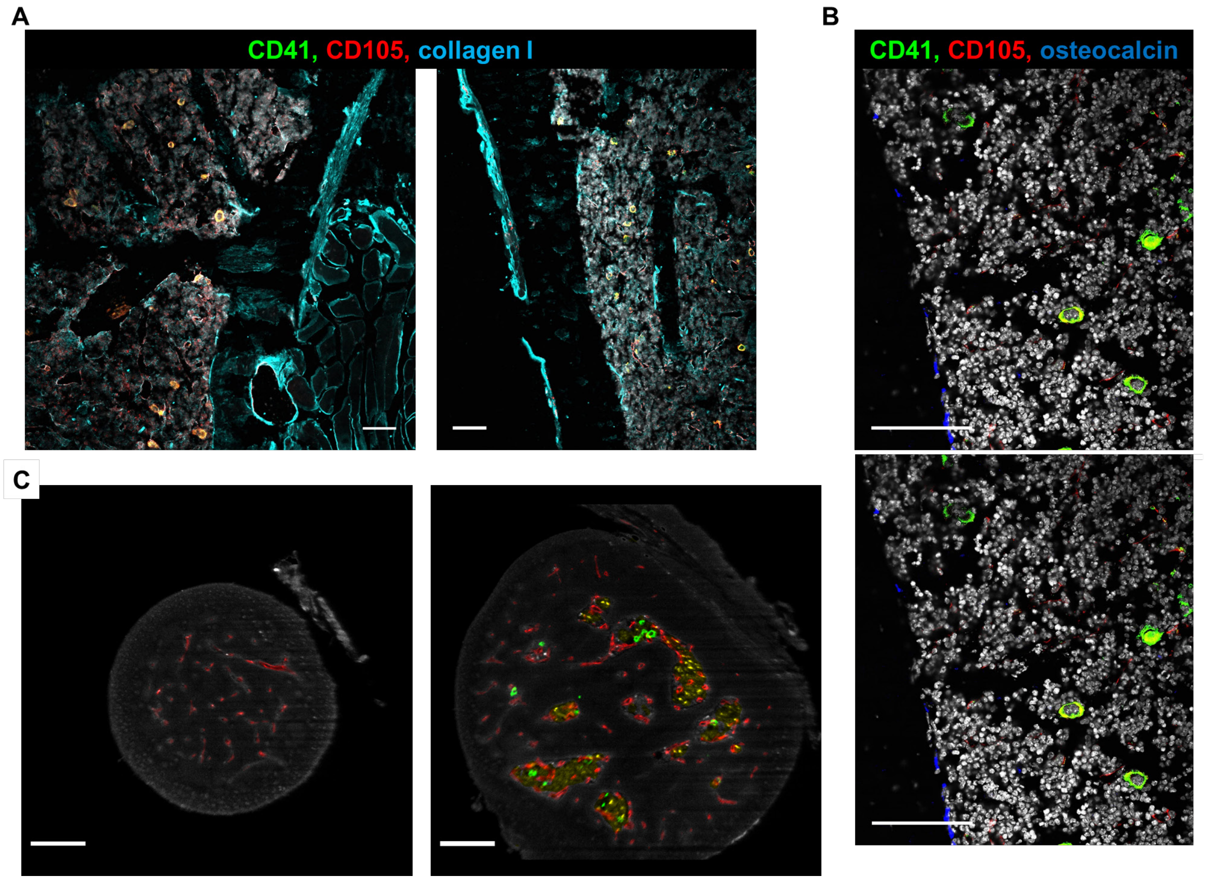

Supplementary Fig. 3. Bone cortex can be identified and used for quantitative analyses. (A, B) Cryo-sections of murine femora were stained for vessels (CD105, red), MKs (CD41, green), collagen I (cyan, only depicted in **A**) or osteocalcin (blue, depicted in **B**). Scale bar 100 µm. **(D)** Different optical z-sections of the Supplementary movie 7 illustrate that the entire BM (yellow pseudo-color based on auto-fluorescence signals) is fully encapsulated in bone (grey). Of note, all BM 'caves' contain blood vessels (CD105, red) and MKs (GPIX, green). Scale bar 200 µm.

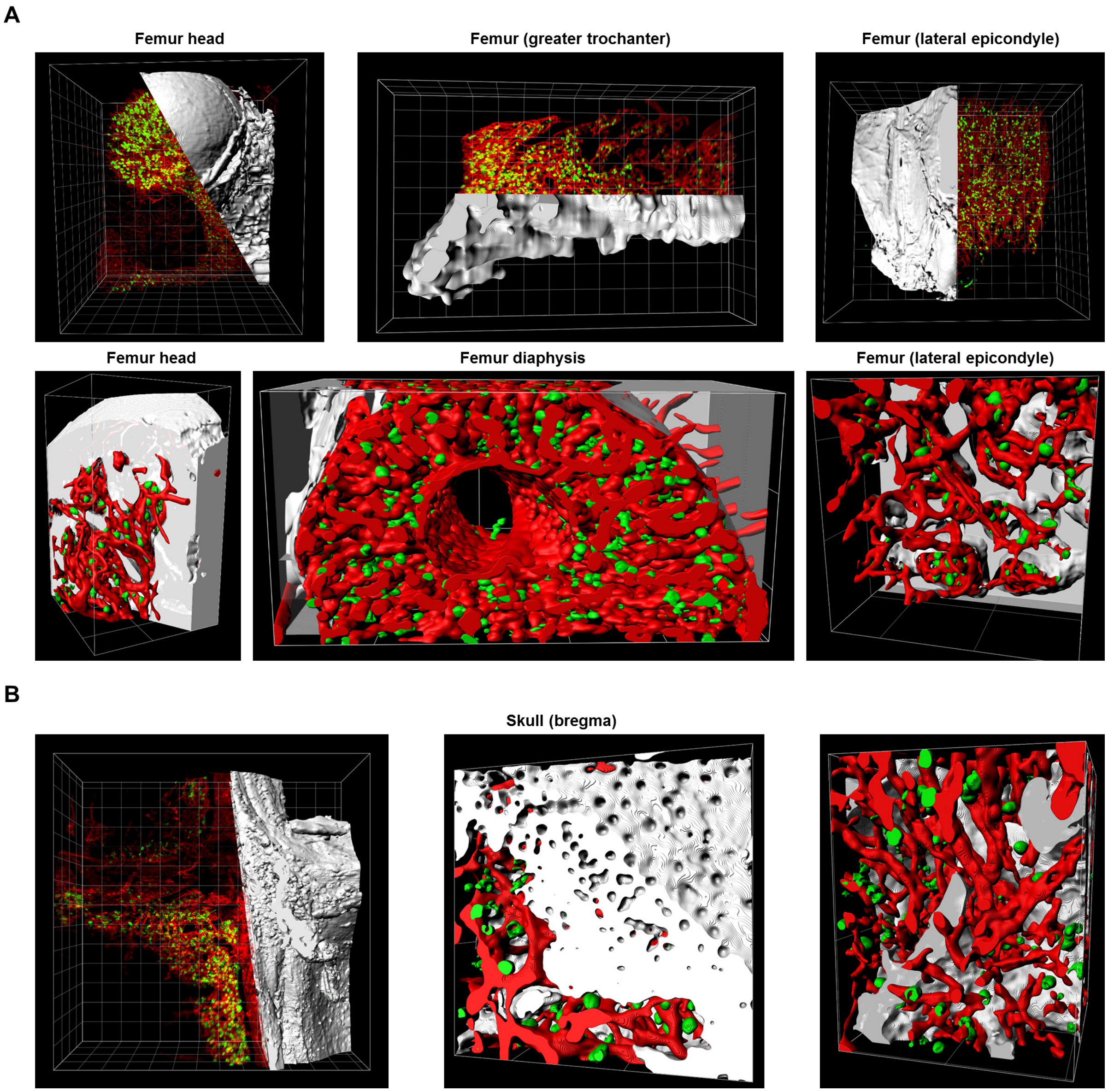

Supplementary Fig. 4. LSFM imaging reveals that the bone cavities are completely filled with MK-containing BM. Optical clearing of intact bones and subsequent LSFM-analysis shows that the entire inter-bone space is filled with bone marrow, containing MKs (green, GPIX) within a dense blood vessel network (red, CD105) in femur **(A)** and cranial BM **(B)**. Unit size for all images: 200 µm.

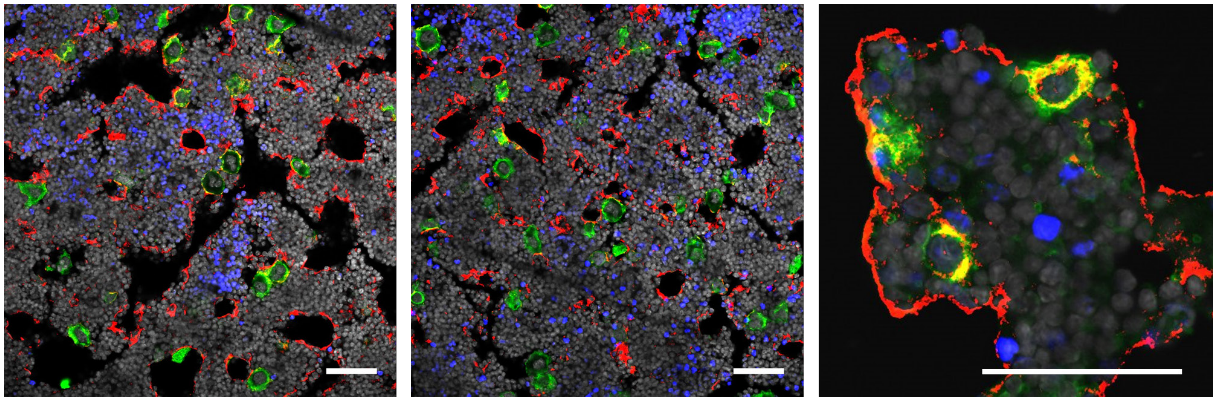

Supplementary Fig. 5. Early EdU-staining is not restricted to the proximity of the vasculature. Femurs were sectioned and stained for EdU (blue), which is incorporated into nuclei (grey). MKs are stained with anti-CD41 antibodies (green) and vessels with anti-CD105 antibodies (red). EdU incorporation was detected in vessel-associated and non-vessel-associated cells. Scale bar 50 µm.
